# Tongue Pressure Sensing Array Integrated with a System-on-Chip Embedded in a Mandibular Advancement Splint

**DOI:** 10.3390/mi9070352

**Published:** 2018-07-14

**Authors:** Yun-Ting Chen, Kun-Ying Yeh, Szu-Han Chen, Chuang-Yin Wang, Chao-Chi Yeh, Ming-Xin Xu, Shey-Shi Lu, Yunn-Jy Chen, Yao-Joe Yang

**Affiliations:** 1 Department of Mechanical Engineering, National Taiwan University, Taipei 10617, Taiwan; ytchen@mems.me.ntu.edu.tw (Y.-T.C.); wang2333@mems.me.ntu.edu.tw (C.-Y.W.); scott@mems.me.ntu.edu.tw (C.-C.Y.); xmx@mems.me.ntu.edu.tw (M.-X.X.); 2 Department of Electronics Engineering, National Taiwan University, Taipei 10617, Taiwan; d98943028@ntu.edu.tw (K.-Y.Y.); sslu@ntu.edu.tw (S.-S.L.); 3 Department of Dentistry, National Taiwan University Hospital, Taipei 10048, Taiwan; schweitzerchen@hotmail.com (S.-H.C.); chenyj@ntu.edu.tw (Y.-J.C.)

**Keywords:** obstructive sleep apnea, mandibular advancement splint, pressure sensing array, conductive polymer, system-on-chip

## Abstract

Obstructive sleep apnea (OSA), which is caused by obstructions of the upper airway, is a syndrome with rising prevalence. Mandibular advancement splints (MAS) are oral appliances for potential treatment of OSA. This work proposes a highly-sensitive pressure sensing array integrated with a system-on-chip (SoC) embedded in a MAS. The device aims to measure tongue pressure distribution in order to determine the efficacy of the MAS for treating OSA. The flexible sensing array consists of an interdigital electrode pair array assembled with conductive polymer films and an SoC capable of retrieving/storing data during sleep, and transmitting data for analysis after sleep monitoring. The surfaces of the conductive polymer films were patterned with microdomed structures, which effectively increased the sensitivity and reduced the pressure sensing response time. The measured results also show that the crosstalk effect between the sensing elements of the array was negligible. The sensitivity of the sensing array changed minimally after the device was submerged in water for up to 100 h.

## 1. Introduction

Epidemiological studies published during the past decade have shown that the prevalence of obstructive sleep apnea (OSA) was, on average, about 22% in men and 17% in women. Some studies have also shown that the prevalence has increased significantly (from 2008 to 2013, a 37% increase for men and a 50% increase for women) [1]. Hypercapnia, hypoxemia, and sleep fragmentation are typical OSA symptoms and are caused by the collapse of the upper airway while sleeping [2]. Patients with OSA are at higher risk of being involved in a vehicle crash due to OSA-related characteristics such as daytime sleepiness, high apnea-hypopnea indices (AHI), and low oxygen saturation levels [3]. Moreover, OSA may increase the possibility of fatal cardiovascular events [4].

Nasal continuous positive airway pressure (CPAP) is the most common treatment for OSA. Although health-related improvements with CPAP have been demonstrated, the inconvenience and discomfort of using CPAP has resulted in relatively low acceptance rate by patients, which limits its clinical effectiveness. In recent years, oral appliances, particularly mandibular advancement splints (MAS), which increase the airway lumen volume by shifting the positions of the jaw and tongue, have been developed as a potential alternative treatment for OSA [5]. While MAS have been demonstrated to be effective for some patients, they may be ineffective for other patients or even result in worse OSA syndromes. Therefore, various research teams around the world have reported studies on the fundamental mechanism and efficacy of MAS therapy for OSA.

In general, the change in the airway lumen volume caused by using MAS can be observed by using tools such as endoscopy [6,7], lateral cephalometric radiography [8,9], fluoroscopy [10,11], computed tomography (CT) [12,13], and magnetic resonance imaging (MRI) [14,15]. In Ref. [6], Ryan et al. studied the correlation between the improvement in OSA symptoms and the changes in pharyngeal dimensions produced by a MAS by videoendoscopy of the upper airway. Mehta et al. [8] conducted a randomized controlled study of MAS for OSA using lateral cephalometric radiography. This study suggests that treatment outcome of MAS or OSA can be predicted by a combination of anthropomorphic, polysomnographic, and radiographic measurements. In Ref. [10], lateral cephalometric radiography and fluoroscopy were demonstrated to be effective at evaluating if the advancement of the mandibular will increase the oropharyngeal airway of a patient. In addition, cine CT was used to investigate the effect of MAS on pharyngeal size and shape [12]. Cross-sectional CT images recorded pharyngeal changes for two cycles of respiration. Sanner et al. [14] presented a method for predicting the efficacy of MAS for OSA by using ultrafast MRI to record the pharynx images for patients during both transnasal shallow respiration and performance of the Muller maneuver. These tools and methods are well-established and capable of acquiring accurate volumetric pharyngeal images. However, some of them are quite expensive or bulky; therefore, the measurements can only be performed in hospitals. As a result, these tools are not suitable for conducting measurement of OSA patients during sleep.

This work proposes embedding a tactile sensing array in a MAS to monitor the position and force of a patient’s tongue during sleep. The sensing device, which was fabricated by micromachining techniques, consisted of a patterned conductive polydimethylsiloxane (PDMS) film and a flexible printed circuit board (FPCB) with an array of interdigital electrode pairs. Multi-wall carbon nanotubes (MWCNT) were employed as the conductive fillers in the PDMS film. Also, microdomed structures were transferred onto the surface of the PDMS film using a nylon membrane filter as the mold. The sensor array was connected to a system-on-chip (SoC) capable of retrieving/storing data during sleep, and transmitting data for analysis after sleep monitoring.

## 2. Device Design and Operational Principles

### 2.1. Sensor Array Principle and Design

**Figure 1:**
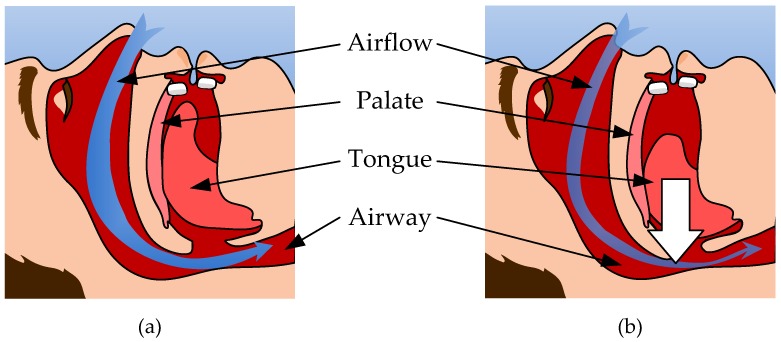
Illustration of the tongue falling back due to gravity. (**a**) Patient’s tongue touches onto palate when sleeping. (**b**) Patient’s tongue falls back due to gravity.

**Figure 2:**
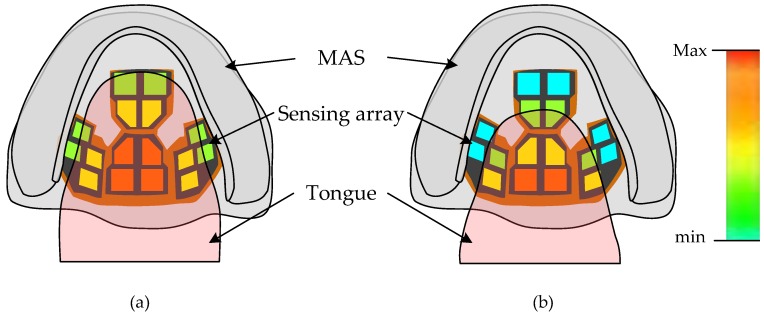
Working principle of device: (**a**) Tongue pressure distribution measured by the sensing array. (**b**) Change of pressure distribution after the tongue falls back.

During sleep, gravity may contribute to the falling back of the soft palate and tongue; this in turn narrows the upper airway and results in OSA symptoms [16], as shown in Figure 1a,b. Therefore, detecting the tongue force imposed on the palate as well as the position of the tongue during sleep may help improve MAS when treating OSA [17]. In this work, the proposed tactile sensing array embedded in a MAS is capable of measuring these physical quantities. Figure 2a illustrates how the pressure force distribution exerted on the palate during sleep can be measured by the proposed sensing array on the MAS. Figure 2b shows that the falling back of the tongue due to gravity in the supine posture can be readily detected by the sensing array.

**Figure 3:**
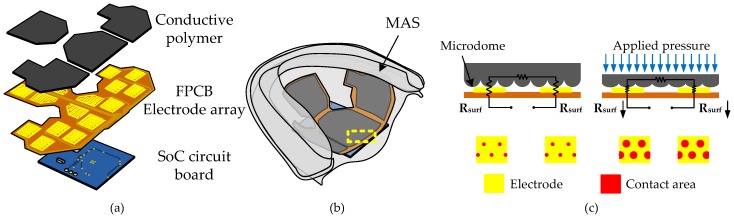
Diagrams of the tongue pressure-sensing device: (**a**) exploded view of the sensing array, (**b**) schematic of the device embedded in a mandibular advancement splint (MAS), and (**c**) cross-sectional view of the sensing element before and after pressure force is applied.

Figure 3a,b shows exploded and schematic views, respectively, of the proposed tongue pressure-sensing device. As shown in Figure 3a, the sensing array consisted of four PDMS-MWCNT polymer films and an FPCB with an array of interdigital electrode pairs (Flex PCB, Ping-Yi Electronics Corporation, Taoyuan, Taiwan). By using a nylon membrane filter as the mold with numerous micropores, microdomed structures were patterned on the surface of the polymer films. These randomly distributed microdomed structures, which have an average base width of approximately 4.5 μm, are essential to achieve high sensitivity for the pressure sensing element. Figure 3b shows the assembled device embedded in a MAS.

Figure 3c shows a cross-sectional view of the contacting interface between the polymer film and the interdigital electrode pair of the FPCB (without the SoC circuit). As uniform pressure is applied, as shown in the right part of Figure 3c, the microdomed structures are deformed, which in turn causes a rapid increase in the contact surface area between the conductive polymer and the planar electrode. Therefore, the contact resistance decreases sharply as the applied pressure increases. This sharp resistance change induced by small external forces is the so-called tunneling piezoresistive effect [18]. Devices with this type of piezoresistive effect are much more sensitive than typical conductive polymer-based devices [19].

### 2.2. SoC Architecture

**Figure 4:**
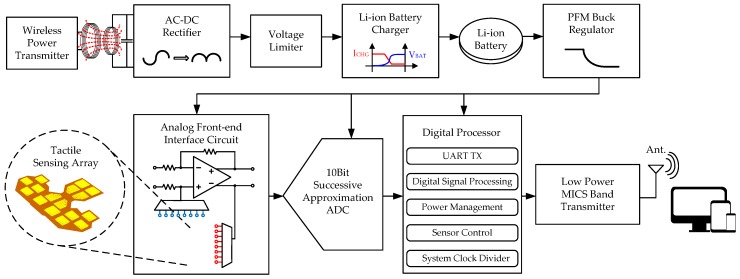
System-on-chip (SoC) block diagram.

The SoC attached to the sensing array is capable of retrieving the detected signal from the sensing array, wirelessly recharging the Li-ion battery, and wirelessly transmitting signals. A block diagram of the SoC is shown in Figure 4. The system consists of an analog front-end (AFE) circuit, a 10-bit successive-approximation-register (SAR) analog-to-digital converter (ADC), a low-power transmitter, and a digital processor, which includes a digital micro-controller unit (MCU) and a power management unit (PMU).

The AFE circuit utilizes a low-power operational amplifier to form a resistor feedback amplifier with a loop gain defined by the ratio of resistors. The SAR ADC is utilized in the proposed system because of its advantages such as low power, high resolution, and low topological complexity. The SAR ADC is only suitable for operating at a relatively low frequency, which is appropriate in biomedical applications. The operation of the SAR ADC is based on a binary-search algorithm, which finds the final interval by comparing the sampled input signal with the voltages of V_ref_/2^*n*^ sequentially.

## 3. Fabrication

### 3.1. Fabrication of the Conductive Polymer

The conductive polymer was a composite of MWCNT and PDMS. The prepolymer for the PDMS-MWCNT composite was prepared by dispersing MWCNT (Golden Innovation Business Corp., New Taipei, Taiwan) with diameters of approximately 20 nm and lengths ranging from 10 to 50 μm into hexane and mixing with PDMS prepolymer (Sylgard 184A, Dow Corning Corp., Midland, MI, USA) at a ratio of 4:1. The mixture was blended with a stirrer for 2 h. The concentration of MWCNT in the PDMS-MWCNT prepolymer was about 6 wt %. Note that the hexane served as the dispersant for improving prepolymer uniformity during blending. Then, the curing agent (Sylgard 184B, Dow Corning Corp., Midland, MI, USA) was dispersed with the PDMS-MWCNT prepolymer at a ratio of 10:1 and stirred for 50 min. After degassing in a chamber for 120 min, the PDMS-MWCNT prepolymer was ready for the subsequent soft-lithography process.

**Figure 5:**
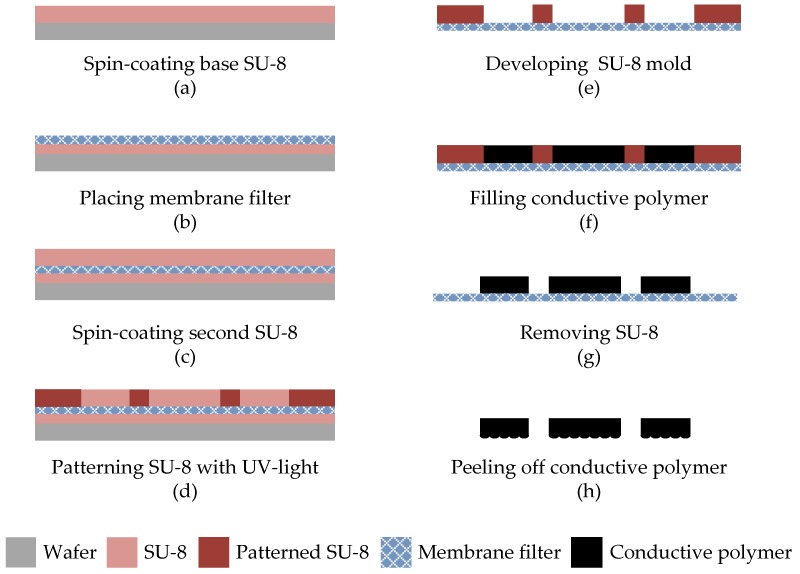
The fabrication process of the conductive polymer with microdomed structures.

**Figure 6:**
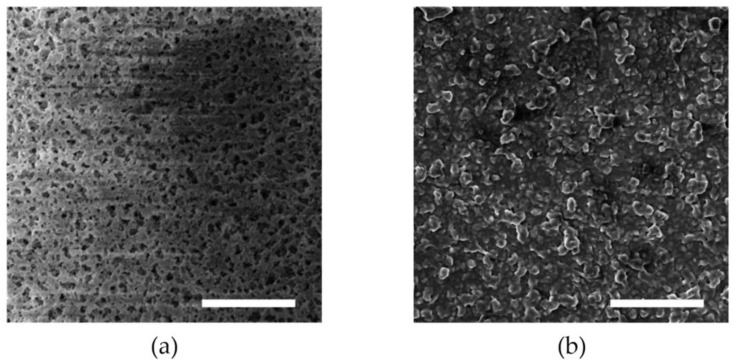
SEM pictures of the (**a**) nylon membrane filter and (**b**) conductive polymer. The scale bars indicate a length of 50 μm.

Figure 5 illustrates the fabrication process of the conductive polymer with microdomed structures. First, a layer of 200-μm thick-film SU-8 photoresist (SU-8 2050, MicroChem Co., Westborough, MA, USA) was spin-coated onto a silicon handling wafer, as shown in Figure 5a. Then, a nylon membrane filter (MS^®^ nylon membrane filter, Membrane Solutions Corp., Kent, WA, USA) with a pore size of 3 μm was placed on the top surface of the SU-8 layer, as shown in Figure 5b. Next, a second layer of 200-μm SU-8 was spin-coated (Figure 5c) and patterned with a dose of 300 mJ/cm^2^ (Figure 5d). As shown in Figure 5e, the SU-8 mold was formed after SU-8 development. Then, a layer of 200-μm PDMS-MWCNT prepolymer was added into the mold (Figure 5f) and cured at 95 °C for 5 h. After removing the SU-8 mold with a stripper (Remover PG, MicroChem Co., Westborough, MA, USA) (Figure 5g), the patterned conductive polymer was peeled from the membrane filter (Figure 5h), and the patterns on the nylon membrane filter were transferred to the conductive polymer. Figure 6a,b show scanning electron microscopy (SEM) images of the surfaces of the nylon membrane filter and the patterned conductive polymer, respectively. The conductive polymer films were then placed to the top of the electrode array on the FPCB. The edges of polymer films were sealed by using PDMS prepolymer.

### 3.2. Assembling and Packaging

**Figure 7:**
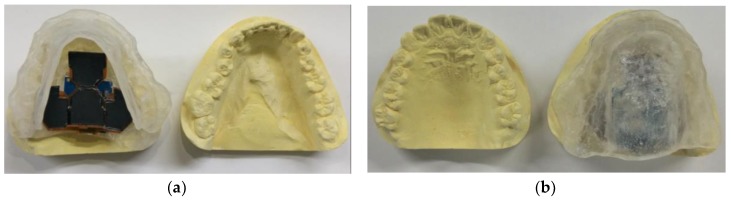
The MAS attached to the (**a**) maxilla mold and (**b**) mandible mold.

Figure 7 shows mandible and maxilla molds on which a MAS was attached. Figure 7a shows the MAS attached to the maxilla mold, and Figure 7b shows the MAS attached to the mandible mold. The proposed device (i.e., the sensing array with the SoC circuit board) was glued to the palatal plate of a MAS using denture resin (Hygenic^®^ Denture Resin, Coltene Corp., Madrid, Spain). The flexible sensing array was assembled with the conductive polymer films with microdomed structures. The shape of the sensing array was designed to allow the sensing array to fit onto the curved roof of the MAS as closely as possible. The sensing array was composed of 16 electrode pairs. Each electrode finger was 400 μm wide, and the gap between fingers was 200 μm. The total sensing area of the interdigital electrode array was 477 mm^2^. The SoC circuit board was connected to the sensing array by soldering a 16-wire flexible flat cable between them. The PDMS prepolymer was applied to the solder spot for both strength and isolation. The entire system was coated with a 3-μm biocompatible Parylene-C film (Parylene-C, La Chi Enterprise Corp., Taipei, Taiwan).

**Figure 8:**
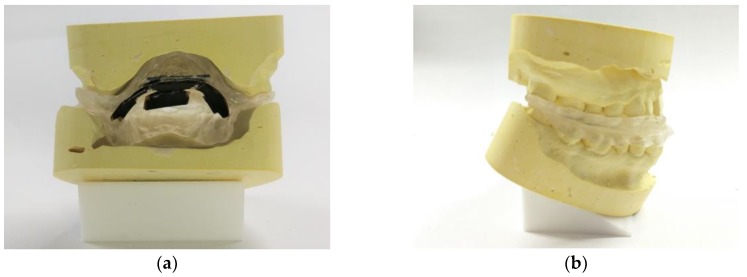
(**a**) Posterior view of the maxilla and mandible molds with the proposed device attached. (**b**) Side view of the molds with the proposed device attached.

Figure 8a shows a posterior view of the maxilla and mandible molds with the proposed device attached. This picture also shows that the flexibility of the proposed sensing array device facilitates its installation onto a customized MAS. Figure 8b shows a side view of the molds with the proposed device attached.

## 4. Measurements

**Figure 9:**
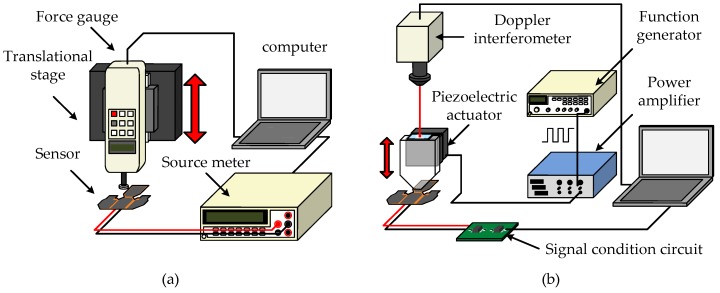
(**a**) Experimental setup for measuring the piezoresistive characteristics of the sensing element. (**b**) Experimental setup for measuring the dynamic responses of the sensing element.

The experimental setup for measuring the piezoresistive characteristics and dynamic responses of the sensing elements are shown in Figure 9a,b, respectively. As shown in Figure 9a, the force applied to the sensing element was measured using a force gauge (HF-1, resolution: 1 mN, ALGOL Instrument Corp., Taoyuan, Taiwan) attached to a vertical translation stage (SR-300C, displacement resolution: 1 μm, Dispenser Tech Corp., New Taipei, Taiwan). A circular polymethylmethacrylate (PMMA) rod of 7.4 mm in diameter was glued on the sensing head of the force gauge. The resistance of the sensing element was measured using a source meter (Model 2400, Keithley Instruments Corp., Solon, OH, USA). Figure 9b shows the experimental setup for measuring the dynamic responses of the sensing element. A piezoelectric actuator (P-621.20L, Physik Instruments Corp., Karlsruhe, Germany) was driven by a power amplifier fed with square wave signals generated by a function generator (GFG8216A, GW Instek, New Taipei, Taiwan). The sensing element was periodically pressed and released by a PMMA block attached to the head of the actuator. The contact area of the PMMA block is 3 mm × 3 mm. A laser Doppler interferometer was employed to measure the displacement of the actuator. The transient behaviors of the sensing element were retrieved using a simple circuit which converted the resistance of the sensing element into a voltage output.

**Figure 10:**
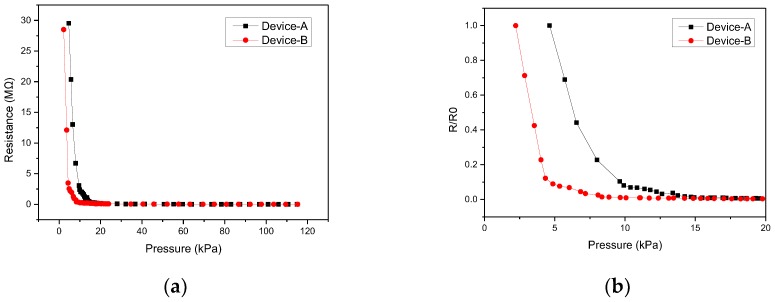
(**a**) Measured responses of the devices of various widths of electrode fingers. (**b**) Normalized measured responses at lower pressure region (0–20 kPa).

**Figure 11:**
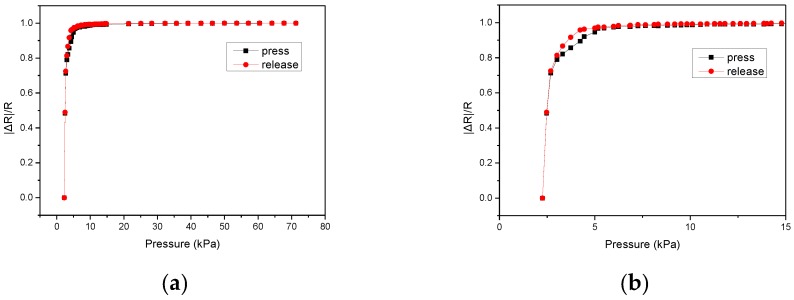
(**a**) Measured hysteresis curve of the sensing element. (**b**) The subset of (**a**) in lower pressure region.

Sensing elements with electrode finger widths of 300 μm (Device A) and 400 μm (Device B) were fabricated and measured. The gap between fingers was 200 μm for both devices. Figure 10 shows the results of the pressure-resistance measurement of the sensing elements. The results indicate that the proposed devices exhibited high sensitivities at lower pressure region (below 10 kPa). It is worth noting that the sensitivity of Device B was larger than that of Device A because Device B had a larger effective electrode area due to its larger finger width. Table 1 shows the sensitivities calculated for devices with various finger widths. Figure 11 shows the measured hysteresis effects of the fabricated sensing element. The external pressure force slowly increased from zero to 70 kPa, and then retreated to zero. As shown in the figure, the hysteresis effect was not significant. It is possibly because the piezoresistive behavior of the proposed sensor is primarily caused by the sharp change in contact area between microdomed structures and the electrodes, which predominates the viscoelastic response observed in typical polymer-based piezoresistive material.

**Figure 12:**
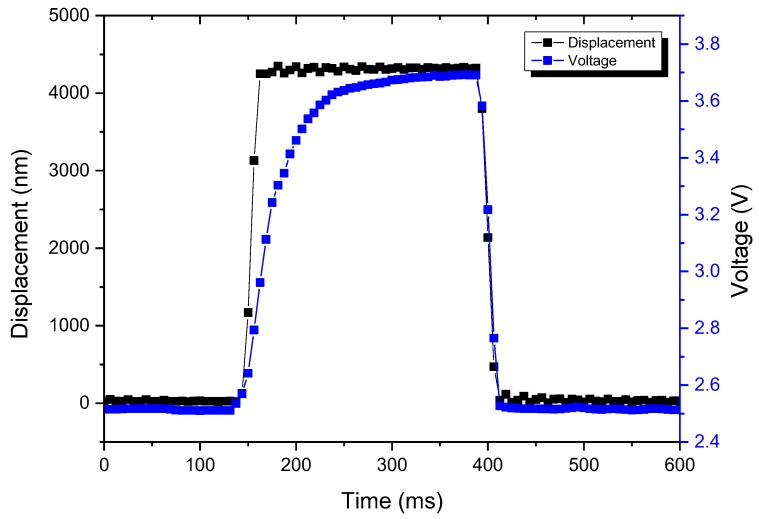
Dynamic square waves response of the sensing element.

**Figure 13:**
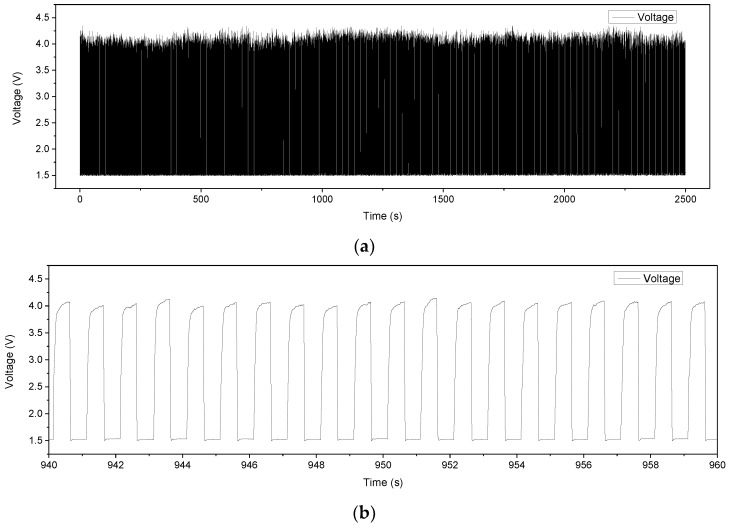
Repeatability test of the sensing element: (**a**) 2500 s of the sensing element’s dynamic responses. (**b**) A snapshot of (**a**) between 940 s and 960 s.

Figure 12 shows the measured dynamic responses of the sensing element as it was periodically pressed and released by the piezoelectric actuator. The sensor response lagged behind the motion of the piezoelectric actuator by about 5 ms. Figure 13 depicts the results of repeatability and drift tests by measuring the sensing element for 2500 cycles using the same experimental setup as shown in Figure 9b driven with a 1-Hz square wave. Figure 13b is the subset snapshot of Figure 13a. The sensing element exhibited reasonably good repeatability and was capable of long-term pressure sensing.

**Figure 14:**
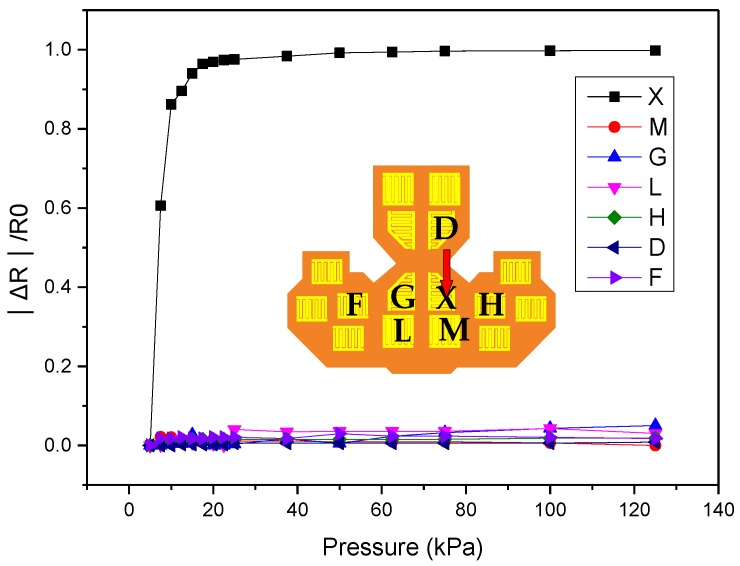
The measured normalized resistances for crosstalk effect measurements. The inset shows the position of the corresponding sensing element on the array.

Figure 14 shows the crosstalk effect measured between the sensing elements. An external pressure force was exerted on point X, and the resistances at points M, G, L, H, D, and F were measured separately. The measured results show that the resistance at point X decreased considerably as pressure force was applied to point X, while the resistances of the adjacent points (i.e., M, G, L, H, D, and F) were almost unchanged even when the applied force was raised to 125 kPa, which indicates that the crosstalk effect was not discernible for the proposed sensing array.

**Figure 15:**
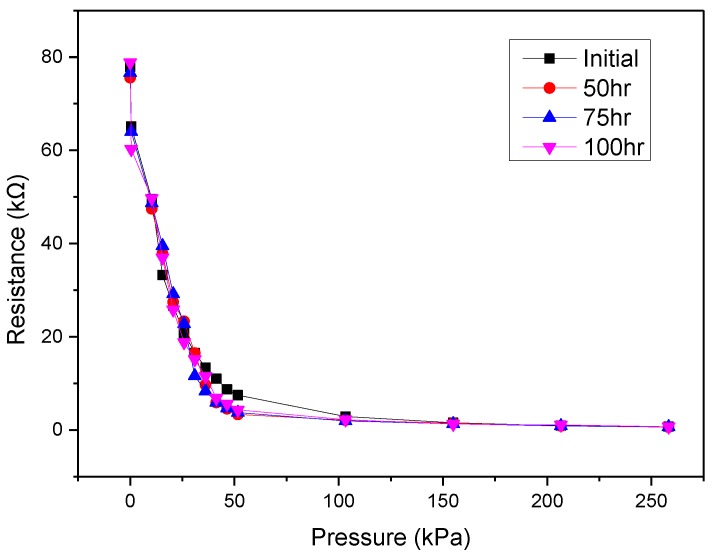
Pressure–resistance curves after the packaged device was submerged in water for different periods of time.

Changes in the proposed device’s sensitivity after the sensing array, which was packaged using Parylene-C, was submerged in deionized water for 50 h, 75 h, and 100 h were evaluated to demonstrate its water-resistant capability. As shown in Figure 15, the pressure-resistance curves of the sensing array after being submerged in water for various periods of time were almost the same as the original one, which indicates that the changes in sensitivity were negligible. This preliminary finding shows that Parylene-C coating can provide reasonably effective short-term water-resistant capability.

**Figure 16:**
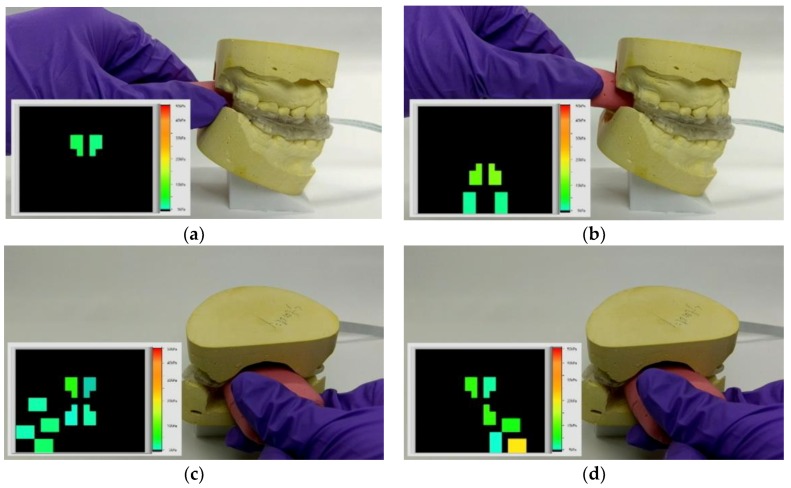
Screenshots of the sensing array measuring the pressure distribution of a silicon tongue. The tongue is pressed against the center (**a**), posterior (**b**), left (**c**), and right (**d**) areas of the sensing array.

Figure 16 shows screenshots of the sensing array’s responses to an artificial silicon tongue touching the sensing array installed on a MAS that was attached between mandible and maxilla molds. The insets in these sub-figures are the pressure distributions measured by the sensing array. The color-bar legend is from 5 kPa (cyan) to 50 kPa (red). Figure 16a,b show the results of when the artificial tongue pressed the center and posterior areas of the sensing array. Figure 16c,d show the results of the tongue pressing the sinistral and dextral areas of the sensing array.

**Figure 17:**
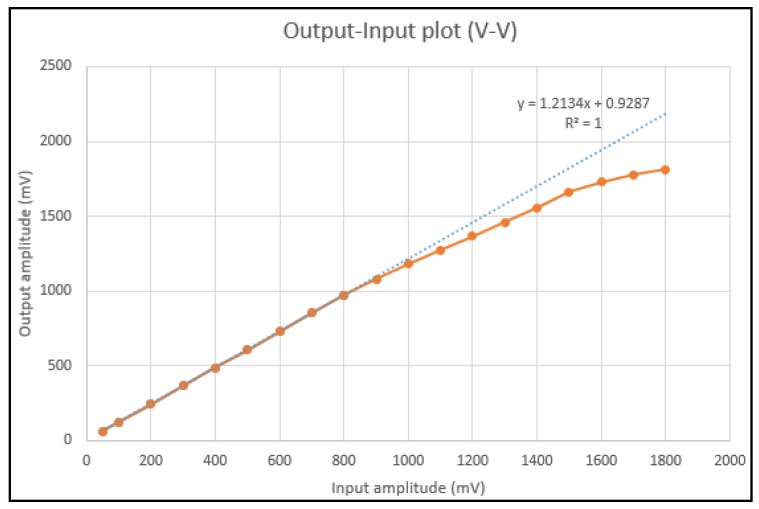
Measured input–output characteristic of the analog front-end (AFE).

**Figure 18:**
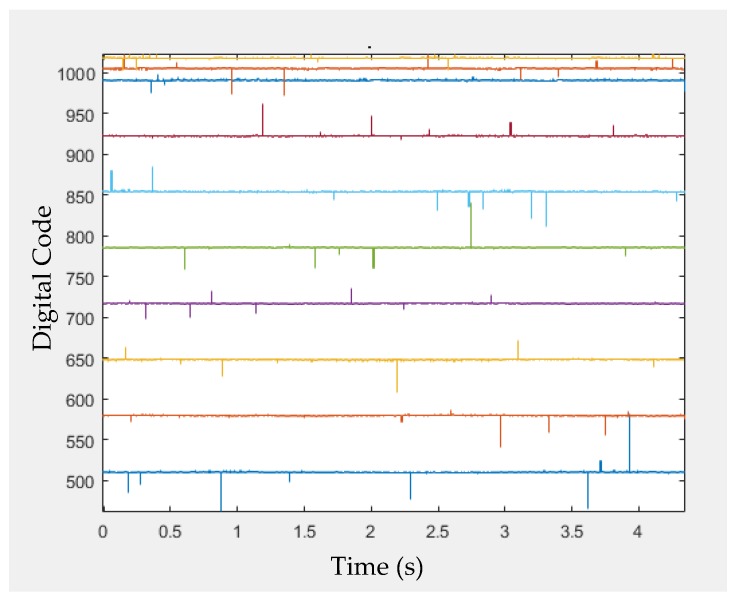
Measured analog-to-digital converter (ADC) output codes corresponding to different DC input levels from the AFE.

**Figure 19:**
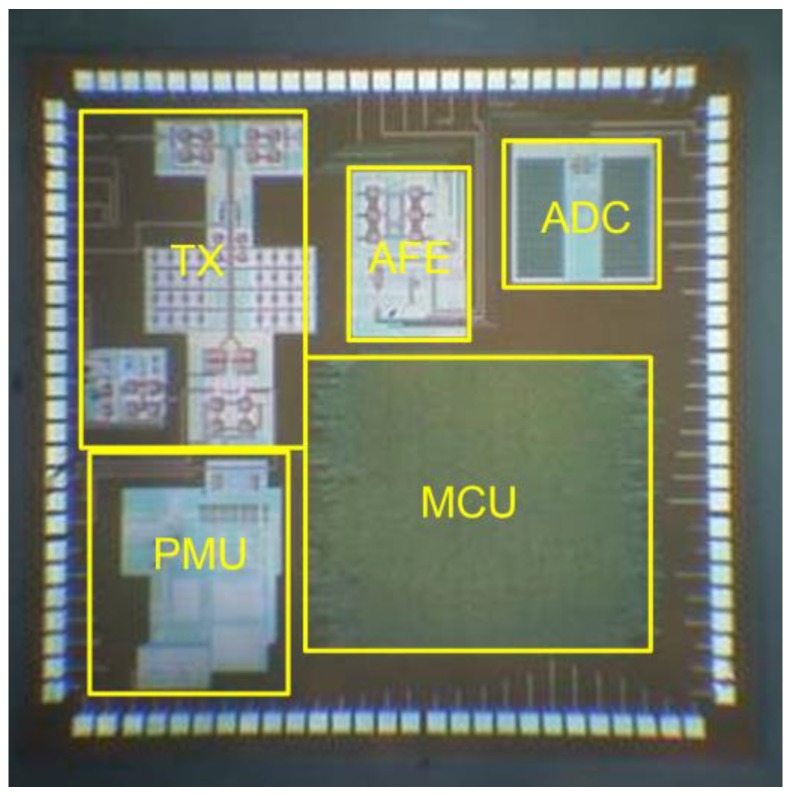
The micrograph of the System-on-Chip for the sensing array.

To achieve a sufficiently compact module fitting in the very limited space of a MAS, a specifically designed SoC chip was realized by UMC 0.18 μm standard CMOS process (United Microelectronics Corp., Hsinchu, Taiwan). Figure 17 shows the measured input-output characteristic of AFE by feeding sinusoidal input signals with amplitudes ranging from 50 to 1800 mV. The blue line is the regression line of small input voltage with a calculated gain of 1.21. The output amplitude starts to be compressed when the input amplitude reaches around 850 mV. Figure 18 shows the measured ADC output codes corresponding to different DC input levels from the AFE. The DC input levels from the AFE were 900, 1000, 1100, 1200, 1300, 1400, 1500, 1600, 1620, and 1640 mV. The corresponding output codes of ADC in Figure 18 presented a quite linear characteristic. The glitches on these curves resulted from supply and environmental noises which can be simply eliminated using additional off-chip components. Figure 19 is the micrograph of the SoC implemented for the sensing array. The chip area, including the testing pads, is 3.16 mm × 3.09 mm. Table 2 lists the measured performance summary which concludes the detailed measurement results of all the sub-blocks in this SoC chip.

## 5. Conclusions

This work presented a highly-sensitive pressure sensing array integrated with an SoC circuit embedded in a MAS for measuring tongue pressure distribution. The proposed flexible device consists of an FPCB with an interdigital electrode array with conductive polymer films and an SoC for retrieving, storing, and transmitting data. The pressure sensing array exhibited high sensitivity and rapid response because of the microdomed structures patterned onto the conductive polymer films. The crosstalk effect between adjacent sensing elements was not obvious. In addition, the device displayed effective short-term water-resistant capability. The proposed device can be used to assist physicians to study and improve the efficacy of MAS for OSA.

## Figures and Tables

**Table 1:** Sensitivities of the devices with different finger widths.

Devices	Device A	Device B
Finger width (μm)	300	400
Gap width (μm)	200	200
Sensitivity (kPa^−1^)	0.177	0.288

**Table 2:** Summary of measured performance of the SoC.

**Summary**	
Technology	UMC 0.18um CMOS
Chip Area	3.16 × 3.09 mm^2^
Supply Voltage	1.8 V
**Analog Front-end**	
SNR	63.1 dB
GainBWProduct	4.4 MHz
Linearity	~0.999
Sensitivity	14 mV/kPa
Power Consumption	6 mW
**SAR ADC**	
Resolution	10 bit
Sampling Rate	200 kS/s
SFDR	74.0 dB@99.8 kHz
DNL	+0.127/−0.18 LSB
INL	+0.30/−0.14 LSB
ENOB	9.47 bit@99.8 kHz
Power Consumption	6.24 mW
**Digital Control Circuits**	
Clock Rate	UART: 38.4 KHz; Scan rate: 1 Hz
Power Consumption	2.53 mW
**Buck DC-DC Converter**	
Input voltage	3.6 V
Power Consumption	6 mW @ 98 mA, η = 78.3%
**OOK Transmitter**	
Carrier Frequency	403 MHz (MICS)
Output Power	−14 dBm
Power Consumption	1 mW
**Off-chip Components**	
Li-ion Battery	3.6 V/50 mAh
Power Consumption	EEPROM: 0.72 mV @ Read; 9 mV @ Write

## Supplementary Materials

Supplementary File 1
